# Healthcare Financing in Saudi Arabia: A Comprehensive Review

**DOI:** 10.3390/healthcare12242544

**Published:** 2024-12-17

**Authors:** Kesavan Sreekantan Nair, Yasir Hayat Mughal, Fahad Albejaidi, Ali H. Alharbi

**Affiliations:** Department of Health Informatics, College of Applied Medical Sciences, Qassim University, P.O. Box 6666, Buraidah 41542, Saudi Arabia; k.nair@qu.edu.sa (K.S.N.); f.alonazy@qu.edu.sa (F.A.); ahhrbie@qu.edu.sa (A.H.A.)

**Keywords:** healthcare, Saudi Vision 2030, health system, challenges, health insurance

## Abstract

Saudi Vision 2030 is a game-changer for all aspects of the economy, including healthcare. This article provides a comprehensive overview of healthcare financing in the Kingdom of Saudi Arabia (KSA). It identifies key healthcare financing challenges that must be addressed to achieve the initiative’s envisioned health system goals. The review also examines and demonstrates how healthcare funds in the KSA are allocated among different healthcare services, to offer a perspective on resource use efficiency at various healthcare levels. This research used a mixed-method design which includes a literature review and secondary data analysis. A literature review was conducted aligned with the Preferred Reporting Items for Systematic Reviews and Meta-Analyses (PRISMA) reporting guidelines. The secondary data were gathered from the reports and websites of government agencies, international organizations, and non-governmental organizations. Despite implementing significant reforms in its healthcare system, the share of private healthcare expenditure in total healthcare spending has seen only marginal growth. The current healthcare financing system appears insufficient to adequately support the chronically ill and the poor. There is a significant imbalance in the allocation of government budgets between hospitals and primary care, with four-fifths of financial resources directed towards hospital services. The Ministry of Health’s budget allocation prioritizes personnel compensation, potentially reducing the available budget for medicines and other essential healthcare supplies. Ongoing reforms in the health sector, including privatization, public–private partnership initiatives, and the government’s commitment to developing a robust primary healthcare network, are expected to play a significant role in controlling rapidly increasing public healthcare expenditures in Saudi Arabia.

## 1. Background

The Kingdom of Saudi Arabia (KSA) is one of the largest nations in the Gulf region, with a population of approximately 34.11 million as of 2021 [1]. According to the World Bank classifications (2021), KSA is categorized as one of the high-income countries [2]. In terms of PPP international $, the country’s Gross Domestic Product (GDP) per capita in 2022 is 59,279, surpassing some of the Organization for Economic Cooperation and Development (OECD) countries like the United Kingdom (54,929), France (55,387), and Canada (58,347) [3]. Its economy largely depends on oil resources, which accounted for about 80% of the government’s revenue in 2022 [4]. Since the launch of the development plan in the early 1970s, KSA has allocated significant resources to the health sector, which have greatly contributed to improved access to healthcare services across the Kingdom. The annual budget allocation to the Ministry of Health (MOH) has also increased significantly, from 2.8% of the total government’s budget in 1970 to 8% in 2022 [1]. Over the years, the population’s health has significantly improved. However, KSA’s health outcomes remain lower than those of its peers. The average life expectancy at birth is 74.3 years, compared to 81.4 years in the United Kingdom, 82.5 years in France, and 82.2 years in Canada [5]. The infant mortality rate of six per 1000 live births is higher than the rates of four in the United Kingdom, three in France, and four in Canada [5]. Similarly, the maternal mortality rate is 16 per 100,000 live births, compared to 10 in the United Kingdom, eight in France, and 11 in Canada [5].

### 1.1. Introduction to Healthcare Financing in the KSA

Healthcare financing aims to mobilize and allocate funds within the health system to meet the healthcare needs of the population [6]. There is no universally accepted approach to healthcare financing, and methods of financing healthcare are influenced by political, cultural, social, and economic factors present in each country [7]. The Beveridge model, developed by Sir William Beveridge (1948) in the United Kingdom, is primarily centered around a national health service [8]. The Bismarck model, developed by Otto von Bismarck, is a decentralized health system model, under which both employers and employees share the responsibility of contributing towards sickness funds through payroll deductions [9]. Currently, Germany, Japan, Belgium, Switzerland, France, the Netherlands, and, to some extent, the USA have implemented this model [10]. The national health insurance (NHI) model is a blend of the Beveridge and Bismarck models [11]. This model is adopted by the health systems in Taiwan, Canada, and South Korea, as well as in the American Medicare system. The out-of-pocket (OOP) model is widely used in many developing countries where resources are lacking to establish a healthcare system similar to the above-mentioned models. This model can be seen in many countries in the Asian and African regions [12]. Each of the above models is unique, and several countries do not strictly adhere to a single model, but they create a combination of strategies that is appropriate for their specific situation.

The healthcare system in the KSA operates under a mixed model that includes both government and private sectors. The major source of healthcare financing comes from the government budget and is mainly derived from the oil sector. The healthcare system relies on three main sources to deliver health services: First, all Saudi nationals and expatriates engaged in the public sector (together with their dependents) are provided with comprehensive healthcare services, free of cost, through MOH hospitals and healthcare centers [13,14]. The second source is private healthcare, which accounts for a smaller but significant share of total health expenditure, is funded through out-of-pocket payments, private health insurance, and employer contributions. Under the Compulsory Employment-Based Health Insurance (CEBHI) program, employers in the private sector must offer health insurance to their employees and their dependents to cover the costs of healthcare in private facilities. The expatriate workforce is utilizing a significant share of the private healthcare sector [15]. The third source of health services is made up of other government ministries and agencies, including the Armed Forces, National Guard, Ministry of the Interior, Ministry of Human Resources, the Royal Commission of Jubail and Yanbu, and public enterprises like ARAMCO, which are also required to deliver health services to their employees and their family members [13]. The MOH serves as the key provider of healthcare services, with 287 hospitals (45,330 beds) and 2121 primary healthcare centers around the country [1]. To serve the needs of their employees and families, other ministries and public agencies manage 51 hospitals (4005 beds), whereas the private sector owns 159 hospitals (17,889 beds) and 3732 medical complexes across the Kingdom [1]. All public health program implementation, policy formulation, and planning, including regulations, are the responsibility of the MOH [13,15].

The KSA and other Gulf Cooperation Council (GCC) countries such as Bahrain, Qatar, Kuwait, Oman, and the United Arab Emirates (UAE) share several cultural, social, and economic similarities. Like the KSA, oil revenues continue to play a crucial role in funding public healthcare services in these countries. However, there are considerable differences in healthcare financing approaches and the extent of private health sector involvement. Bahrain and Oman provide free or highly subsidized public healthcare services, but both have embarked on initiatives to introduce health insurance for expatriates and to slowly increase the private sector’s role in financing and delivering healthcare services [16]. Qatar has a largely publicly funded healthcare system, and the proposed universal health insurance is expected to increase cost-sharing and integration with the private sector [17]. Kuwait provides healthcare services free to its citizens; however, its public–private partnership program aims to manage healthcare costs and improve access to healthcare services [18]. The UAE healthcare system combines public and private funding with mandatory health insurance in Dubai and Abu Dhabi [19].

In terms of GDP, the aggregate healthcare expenditure in the KSA increased from 4.4% of GDP in 2001 to 5.97% of GDP in 2021 [20]. The Kingdom spends a larger share of its GDP on healthcare among the GCC countries. Its public expenditure on healthcare constituted 76.98% of healthcare expenditure in 2021 [20]. This figure is higher compared to Bahrain (65.9%) and the UAE (64.1%), but lower compared to Kuwait (89.7%), Oman (87.6%), and Qatar (85%). Out-of-pocket expenditure (OOPE) contributed 10.16% to current healthcare expenditure (CHE), while private healthcare expenditure made up 23.2% [20].

### 1.2. Historical Context

Traditionally, Saudi Arabia has provided free healthcare services to its citizens at the point of delivery. The Kingdom’s strong oil-based economy has enabled the government to offer universal access to healthcare services, including to expatriates. However, this policy has faced several challenges, such as escalating healthcare costs, demographic transitions, an aging population, changing lifestyles, shifting disease patterns, and inefficiencies in healthcare management [13,14,21,22,23,24,25]. Furthermore, the delivery of free health services to the huge expatriate population that constitutes about one-third of the KSA’s population is another major challenge. Despite the significant resources that the KSA can allocate to healthcare, it faces a financial burden due to these challenges, leading to a rapid increase in healthcare expenditures [25,26]. To manage these challenges, the KSA has implemented healthcare financing reforms with an overall goal of reducing the burden on the government budget and meeting ever-increasing healthcare demands. As part of these reform measures, the KSA introduced CEBHI in 1999, which was implemented in phases for all workers in the private sector, with premium contributions from their employers [27]. CEBHI marked the beginning of private sector participation in financing healthcare services in the KSA. By expanding health insurance for expatriates and eventually extending it to Saudi nationals in the private sector, the reform encouraged a transition towards collective financial responsibility. The program, which currently covers over 11 million Saudi nationals and expatriates, has had a significant impact on the country’s healthcare financing system [28].

### 1.3. Current Challenges

Despite significant progress in its healthcare landscape, the KSA faces many challenges. First, its healthcare services have mainly been financed by oil revenues, with services offered free at the point of delivery. However, variations in oil prices and rising healthcare expenditures have raised concerns regarding the long-term sustainability of this model [29]. A continuous rise in development expenditures has resulted in budget deficits over several years. From 1980 to 2017, there were 22 years of budget deficits, varying from 2% to 25% [30]. However, there were also 13 years of budget surpluses during this period, ranging from 1% to 32% [30]. To achieve a stable economy with a balanced budget, the government implemented significant reforms, such as decreasing subsidies to various sectors, cutting government expenditures, applying fiscal corrections, and channeling non-oil resources into a balanced budget [13].

Second, healthcare expenditure in the KSA is further poised to increase due to factors such as demographic transitions, increasing lifestyle diseases, and the increasing demand for quality healthcare [13]. Third, there are challenges in achieving Universal Health Coverage (UHC) and ensuring equitable access to healthcare services across rural and urban regions and different segments of the population, including expatriates [13]. Fourth, despite many preventive care efforts, NCDs such as heart diseases, cancers, and diabetes continue to present significant health challenges. Fifth, despite increased healthcare spending, public healthcare services face issues that warrant the need for reforms, such as differences in service quality, long waiting times, and inefficiencies in resource allocation [13]. Finally, the KSA also faces the issue of retention of highly skilled healthcare professionals in rural and remote regions. This issue requires more realistic planning for health workforce development and long-term strategies.

### 1.4. Saudi Vision 2030 and Health Sector Transformation

Saudi Vision 2030, launched in 2016 by Crown Prince Mohammed bin Salman, is an ambitious plan to overhaul both the economy and society [31]. This is a strategic plan of action that aims to reduce the reliance on oil resources and diversify the economy through innovative, revolutionary reforms to the country’s economic and social systems [31]. The emphasis of the vision is on strengthening the financing and provision of the healthcare delivery system to achieve UHC. The theoretical foundation of the vision is rooted in a few fundamental principles [31,32]:

∘ Diversification of revenue: As the KSA has historically been dependent on oil revenue, the Vision aims to decrease this reliance by encouraging expansion in non-oil sectors. This diversification is crucial for ensuring sustainable growth and the protecting the economy from global oil market fluctuations.
∘ Market liberalization: The vision adopts a neoliberal economic model, with a focus on market liberalization, deregulation, and privatization. This method seeks to attract foreign investment, in turn promoting a competitive economic atmosphere. The plan incorporates strategies that promote the involvement of the private sector and decrease economic reliance on the government.
∘ Sustainable Development Goals: Following international sustainable development goals, the Vision emphasizes social welfare, environmental sustainability, and economic growth. The plan involves methods to enhance citizens’ quality of life, improve public services, and encourage social responsibility.
∘ Strategic partnerships and global integration: The Vision focuses on creating strategic alliances at both local and international levels to strengthen economic cooperation and knowledge sharing. This involves initiatives to integrate the Kingdom into the global economy via trade.

The Health Sector Transformation Program (HSTP), a long-term roadmap developed as part of Vision 2030, aims to reform the health sector into a comprehensive, integrated, and effective system focused on the well-being of the individual and society [32]. The key objectives of the HSTP are improved access to healthcare, improved quality and efficiency, prevention of diseases, and enhanced digital healthcare services [32]. The HSTP is founded on the value-based care philosophy, which ensures financial sustainability and transparency through promoting public health, combating illnesses, and adopting a new model of care that focuses on disease prevention. While guaranteeing comprehensive coverage and equitable geographic distribution, the program also aims to expand the accessibility of e-health services and improve the overall quality of healthcare services.

The Vision has made considerable leaps in transforming the healthcare sector in the KSA. It is paving the way for an equitable, efficient, and patient-centered healthcare system. Economic diversification is one of its main objectives. Since the launch of Vision 2030, the non-oil sector has been growing; according to the International Monetary Fund (IMF), this sector grew by 4.8% in 2022 [33]. This expansion has been fueled by investments in technology, entertainment, and tourism. In the sphere of privatization, government-driven programs like the Public Investment Fund (PIF) have boosted capital influx into developing sectors. Since 2018, the Vision 2030 framework has led to a 13% yearly increase in Foreign Direct Investment (FDI), boosting the growth of non-oil sectors [34]. The integration of digital health solutions led to a tremendous increase in teleconsultations during the COVID-19 pandemic, showing resilience in response to disruptions in healthcare services [35]. In 2023, approximately 1.6 million virtual consultations and 9.2 million virtual appointments were conducted [36], with nearly 36% of all appointments and consultations taking place virtually [36]. In the field of medical education, collaborations with global medical institutions have resulted in an increase in specialized medical professionals since 2018. Notably, the KSA launched SEHA, the largest virtual hospital, connecting more than 150 hospitals globally and offering 30 highly specialized healthcare services [31]. Additionally, the establishment of a national guidelines center to promote evidence-based practices and the use of digital health technologies to improve access and quality of healthcare [37] further highlight the KSA’s commitment to leveraging digital technology for enhanced healthcare access across different segments of the population.

### 1.5. Need for a Comprehensive Review

Since the adoption of Vision 2030, there has been a growing body of literature examining various aspects of healthcare financing in the KSA. These studies address a wide range of topics, including the challenges of the CEBHI program [27], healthcare financing and the financial sustainability of the health system [38,39,40,41,42], the OOPE burden among the population [43,44,45,46], the role of health insurance in reducing OOPE [46], OOPE and health-related quality of life [47], resource allocation in government hospitals [48], the economic burden associated with chronic diseases [49,50,51], the role of health insurance in households’ health-seeking behavior [52], health insurance knowledge and its benefits among insured people [53,54], fraud and abuse in health insurance [55], households’ willingness to pay (WTP) for health insurance [56,57,58,59,60,61], policy options for financial sustainability [62], health funding models [63], and the national health accounts (NHA) framework [64]. In addition, there are few studies that focused on privatization and public-private partnerships in healthcare [65,66,67,68,69].

The healthcare financing system in the KSA is undergoing significant reforms, driven primarily by the goals outlined in Vision 2030. Conducting a thorough analysis of the nation’s healthcare financing system would offer a comprehensive understanding of its strengths, weaknesses, and the challenges the country faces in moving towards universal coverage. Allocation of resources for healthcare is a critical process that determines how financial resources are distributed across various components and levels of care, from primary care to specialized hospital services. An analysis of resource allocation provides critical information to healthcare policymakers to assist in the judicious allocation of healthcare resources and achieve allocative efficiency. This research seeks to provide a comprehensive overview of the current state of healthcare financing in the KSA and identifies key challenges that need to be addressed in achieving the healthcare system goals set forth by the Vision 2030 initiative. This research also intends to examine and demonstrate how funds are allocated among different healthcare activities, to offer a perspective on the efficiency of resource use at various levels of healthcare.

## 2. Materials and Methods

This research used a mixed-method design, which includes a literature review and secondary data analysis. The literature review method was applied to stimulate pertinent descriptive information on various aspects of healthcare financing in the KSA. The authors collected relevant information about different aspects of healthcare financing through studies published in peer-reviewed journals. Secondary data collated from various sources were analyzed and used. These include official documents of Saudi Vision 2030, the Health Sector Transformation Program (HSTP) developed by the MOH, annual statistical books of the MOH, National Health Accounts Estimates (NHA) produced by the Saudi Health Council, annual reports of the Saudi Arabian Monetary Agency (SAMA), and other relevant policy documents of the government. Relevant data were also used from reports, documents, and websites of the World Bank, the World Health Organization (WHO), the International Monetary Fund (IMF), the Organization for Economic Cooperation and Development (OECD), and non-governmental organizations.

The review of the literature was conducted in line with the Preferred Reporting Items for Systematic Reviews and Meta-Analyses (PRISMA) reporting guidelines using databases such as Google Scholar, Science Direct, PubMed, and Scopus. The authors searched the research articles published in the English language between 2005 and 2022, as this period was considered more appropriate and representative of the changes in the healthcare financing scenario in the KSA, before and after the launch of Vision 2030. Moreover, Saudi Arabia began rolling out CEBHI for private-sector workers in 2005. This marked a significant shift in healthcare financing, introducing a new funding source beyond government revenues. By focusing on this period, researchers can capture significant changes and reforms in the KSA’s healthcare financing landscape. The search was conducted during the period between 25 March and 30 April 2023. The search terms used were health finance OR healthcare finance OR health insurance OR out-of-pocket expenditure OR health expenditure OR health spending OR healthcare spending AND Saudi Arabia OR Kingdom of Saudi Arabia.

**Figure 1:**
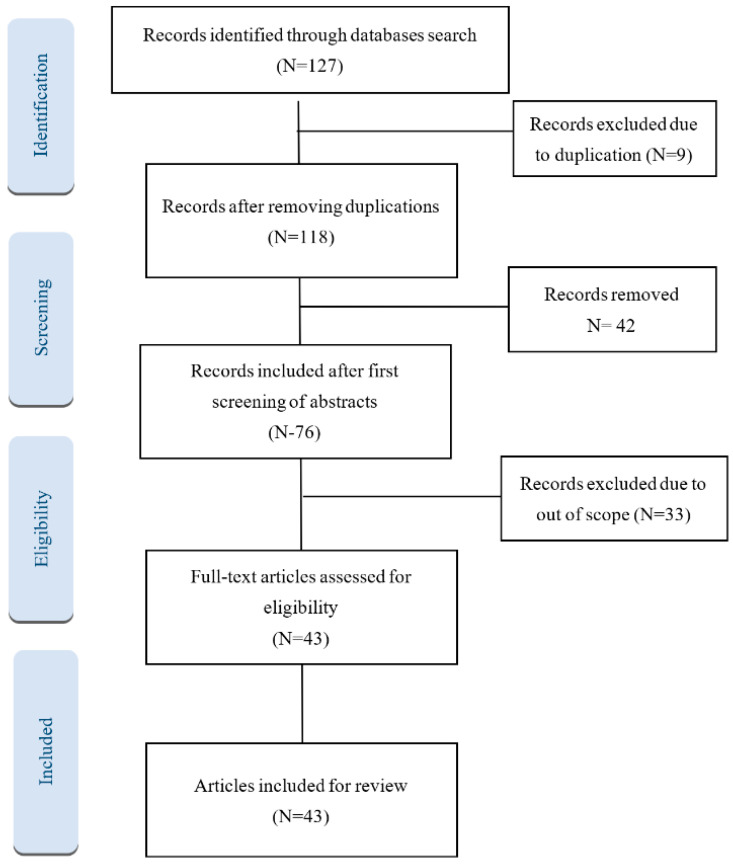
Flow diagram based on guidelines of PRISMA.

Key criteria used to select studies included those relevant to healthcare financing in the KSA, studies that used qualitative, quantitative, or mixed-methods approaches, studies that included topics related to healthcare financing, health insurance, out-of-pocket expenditure, financial protection, and studies that examined multiple aspects of healthcare financing systems such as revenue collection, pooling of funds, and purchasing of services. The preliminary search strategy by the authors facilitated the identification of 118 scientific articles. However, further screening of the papers and documents through the review method resulted in the inclusion of 43 articles (Figure 1).

Two authors performed the initial screening of articles based on titles and abstracts, and the authors arrived at a mutual agreement regarding relevance for inclusion. As there is a certain overlap of reporting among databases, many duplicate articles were dropped. Articles without abstracts in English, not related to Saudi Arabia, and unrelated to healthcare financing were excluded. The criteria for screening the abstract were also applied to the full-text screening of the articles. Once the title and abstract were screened, the data from the full text was extracted and double-checked, and key themes were extracted. While reviewing the selected studies, the authors revisited and clarified the predetermined inclusion and exclusion criteria to ensure everyone had a shared understanding of the selection parameters. Each author presented the reason for including or excluding specific studies, allowing others to arrive at a common consensus.

Secondary data collected from various documents and websites were categorized by health expenditure, such as government health expenditure, private health expenditure, and out-of-pocket expenditure. Using NHA data, the contribution of each healthcare financing scheme in health services delivery and the allocation of health funds to various levels of care are presented. The data for the most recent available year are reported and shown with the help of statistical tables and figures. Expenditure data are reported in U.S. dollars using an average exchange rate for the year as reported by the SAMA.

The authors conducted the literature review and secondary data analysis simultaneously. The integration of both approaches allows the authors to leverage existing data while grounding their analysis in the current literature. The interactive process of moving between literature review and secondary data analysis provides deeper insights and robust conclusions. The insights from both the literature review and secondary analysis help to develop a comprehensive understanding of the topic. All related information and data thus consolidated from various sources were used by way of triangulation to give more integrity to the analysis and discussion.

The key terms used in the analysis and their definitions [70,71] are given below:

- Current health expenditure—Comprises all services such as curative care including services provided to residents by non-resident providers, rehabilitative care, prevention, public health, and ancillary healthcare services. Also includes expenditures for the administration of these services and drugs, medical goods, and salaries and fees of health professionals.
- Financing schemes—Refers to the components of a country’s healthcare financing system that channel revenue to purchase and organize activities within the health accounts boundary
- Healthcare functions—Refers to the goods and services provided, and the activities performed within the health accounts boundary.
- Healthcare providers—Comprises entities that receive funds in exchange for, or in anticipation of, activities inside the health accounts boundary.
- Out-of-pocket expenditure—Refers to direct outlays of households, including gratuities and payments in-kind, made to healthcare providers and suppliers of pharmaceuticals, therapeutic appliances, and other goods and services whose primary intent is to contribute to the restoration or enhancement of the health status of individuals or population groups. This also includes household payments to public services, non-profit institutions, or non-governmental organizations.

## 3. Results

### 3.1. Aggregate Healthcare Expenditure in KSA

A comprehensive estimate of aggregate healthcare expenditure in the KSA was unavailable in the public domain, until NHA estimates for the year 2018 were released by the Saudi Health Council in 2020 [70]. According to the NHA report, overall healthcare expenditure in the KSA (excluding capital formation) was SAR 169.55 billion (US$ 44.15 billion) in 2018, which includes spending by the public (70%) and the private (30%) sectors, both together accounting for 6.4% of the GDP [70]. While households’ OOPE constituted 15.9% of CHE, compulsory health insurance schemes implemented through the private health sector accounted for 12%. About 53.5% of private healthcare expenditure was incurred in the form of OOPE by households, and the remaining 46.6% by employers and corporate sector [70].

### 3.2. Trends in Healthcare Expenditure

**Figure 2:**
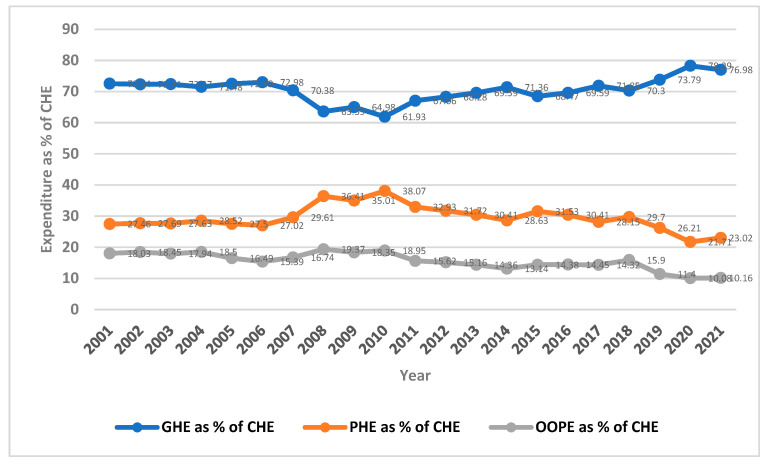
Proportion of government, private, and OOP expenditures as a percentage of current health expenditure (2001–2018). Source: World Bank database.

The healthcare financing trend in the past decade shows there has been a steady increase in government healthcare expenditure, and its contribution to overall healthcare expenditure has increased since 2010 (Figure 2). However, there was a marginal decrease in private healthcare expenditure share from 35% to 23.72% between the period 2010 and 2021 [20]. The proportion of private healthcare expenditure to CHE is likely to increase with the growth in private-sector employment. Moreover, the policy of Saudization has been accelerated, and the percentage of Saudi nationals employed in the private sector has shown a marginal rise from 20.4% in the first quarter of 2020 to 22.8% in the first quarter of 2022 [72]. During the period between 2010 and 2021, the share of households’ OOPE on healthcare decreased from 18.35% to 10.16% [20].

Healthcare expenditure in the KSA is expected to rise further due to factors such as demographic transitions, increasing lifestyle diseases, the escalating cost of health services, and the increasing demand for quality healthcare. There is a higher prevalence of NCDs in the KSA, and treatment costs associated with these diseases consume the largest share of the national health budget, placing a persistent burden on the government resource envelop [49,50,51]. The population of the country is projected to reach 39.5 million by mid-2030, with an aging population and an increase in lifestyle disorders, including related co-morbidities, driving further growth in healthcare demand [32]. By 2035, it is projected that 44% population will be over 40 years of age, and 14% will be above 60. This shift will generate more demand for healthcare, particularly for the treatment of NCDs, placing enormous pressure on government funding for healthcare [8]. By 2035, the KSA will require 20,000 more beds to keep up with these changes [73]. However, based on the global bed density average, the KSA will face a shortage of 40,000 hospital beds [74]. Furthermore, the number of older people (60–79 years) is likely to rise from 1.96 million (2018) to 4.63 million (2030), and there will be 1.60 million people over the age of 80, or roughly 4% of the population of the KSA, which will result in a massive increase in healthcare expenditure [74,75].

### 3.3. Healthcare Financing by Sources

#### 3.3.1. Public Healthcare Financing

In 2018, general government expenditure was estimated at SAR 119.2 billion or US$ 31.78 billion (about 70%), which was incurred through the MOH and other government ministries/agencies [70]. A major share of government expenditure was directed through the MOH, while other government ministries and agencies allocate funds for health services from their respective budgets. Although the information on budget allocation to the MOH is available in the public domain, the details of the budget allocated for healthcare by other government ministries and agencies for their employees and dependents are unavailable. NHA estimates reveal that two-thirds of the CHE is directed towards hospital services and drugs, with government hospitals receiving the largest share at 79% [70]. This contrasts with the average benchmark of 38% for OECD countries [76]. Evidence shows that a balanced allocation of funds between hospital and primary care will enable greater value for money invested in healthcare services [6]. The KSA’s healthcare needs are influenced by a combination of factors, including high rates of chronic diseases, workforce challenges, and unique demographic trends [63]. NCDs alone cause 73% of all deaths, 37% due to cardiovascular diseases and 10% caused by cancers [77]. This major shift from communicable to chronic diseases necessitates a robust primary healthcare system focused on the prevention and management of these conditions [31,32].

Over the past decade, the budget allocated by the Ministry of Finance to the MOH has increased significantly, growing from SR 35,063 million (US$ 9351 million) in 2007 to SR 80,752 million (US$ 21,537 million) in 2023 [78]. This allocation currently represents 7% of the national budget (Table 1). In addition to the MOH budget, the government allocation to the health sector through five-year economic development plans has played a significant role in building up health resources across the country.

**Figure 3:**
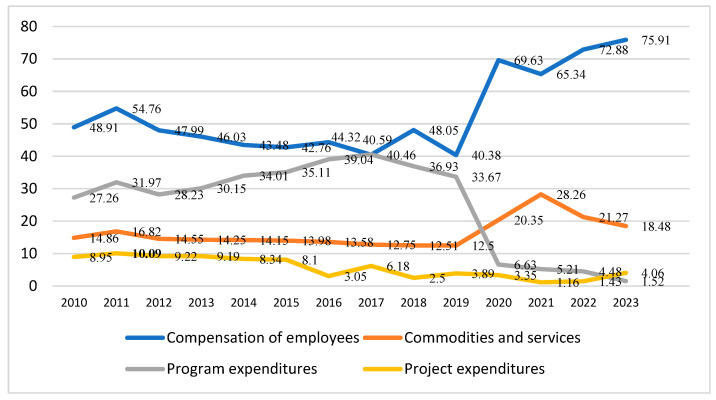
Appropriations for MOH budget 2010–2023 (in %). Source: Health Budget, MOH (KSA).

The MOH budget is distributed across various sub-categories, including compensation of employees, purchase of commodities and services, programs, and projects. A trend in MOH budget allocation to the various sub-categories shows that the percentage of budget allocated to the compensation of employees has been increasing consistently since 2019 (Figure 3).

It is likely that ‘Saudization’ in the health sector has resulted in an increasing percentage of Saudi citizens in the health workforce, who receive a higher salary and allowances compared to the expatriate health workforce [79]. The MOH data shows that the percentage of Saudi doctors (including dentists) in MOH hospitals increased from 21.6% in 2010 to 54.86% in 2023, while the share of Saudi nurses increased from 48.7% to 61.13% over the same period [79,80]. However, the share of program expenditure has decreased drastically. The program expenditure comprised various sub-categories under the general program, maintenance, and self-employment program [78]. The general program includes treatment, replacement, and other programs. The treatment category includes the cost of treatment in the Kingdom and abroad. The replacement program includes the cost of medical and non-medical replacements, whereas the self-employment program covers both existing and new programs [78].

#### 3.3.2. Private Healthcare Financing

NHA estimates show that SAR 50.35 billion (US$ 13.43 billion) was incurred as private healthcare expenditure in 2018, which mainly includes OOPE by households (53.5%) and health insurance contributions (40.8%) by employers [70]. The past trend in private healthcare expenditure based on the World Bank estimates suggests that CEBHI has made a significant impact on healthcare expenditure patterns in the KSA, with a sharp decline in OOPE [20]. In the initial stages of CEBHI, between 2006 and 2008, employers’ contribution towards health insurance increased marginally and, as a result, household OOPE decreased. However, the increase in health insurance contributions was reported as much lower than expected, primarily due to factors such as slower growth of the private sector, an unclear strategic vision, and an insufficient number of service providers [81]. Other reasons include delays in the renewal of work permits and resistance from Islamic scholars [82]. Nonetheless, the enactment of the regulations of cooperative health insurance law in 2014 has brought phenomenal changes in the health finance scenario across the country [41].

Over the last few years, health services utilization in the private sector has increased due to increasing worker enrollment in CEBHI, and in 2021, about 63.14 million outpatient visits and 1.10 million inpatient admissions were reported in private hospitals, compared to 61.95 million outpatient visits and 1.37 million admissions in the MOH hospitals and primary healthcare centers [1]. The increased utilization of private healthcare services may eventually result in a rise in private healthcare expenditure.

#### 3.3.3. Out-of-Pocket Healthcare Payments

Before the establishment of health insurance, OOPE and health insurance contributions from larger enterprises were the primary funding sources for private healthcare. The government hospitals were overloaded and overcrowded, which forced people to utilize health services from the private sector [13]. Saudi citizens who could afford private hospital care sought treatment from private providers, and expatriate workers employed in larger organizations were given the option of voluntary health insurance by their employers. OOPE accounted for about 30% of the KSA’s total healthcare spending in 1995, which further increased to about 48% in 1998 [82]. However, healthcare financing reforms implemented by KSA, particularly the institution of CEBHI, have profoundly altered the KSA’s healthcare financing scenario.

Currently, households’ OOPE on healthcare is estimated to be nearly 10% of total healthcare spending [5]. About 56% of the workforce who are expatriates accesses health services through private health insurance and OOPE [53]. Existing evidence reveals that the presence of chronic diseases is a significant factor contributing to the increase in OOPE on healthcare [43,44,45,46,47]. Studies conducted in the KSA suggest that OOPE is concentrated among the poor, the elderly, those with low levels of education, and those suffering from chronic diseases [43,44,45,46]. These groups tend to incur a significant amount of OOPE while seeking treatment in the private sector, highlighting the inadequacy of current healthcare financing strategies in lessening the economic burden, especially for low-income populations.

#### 3.3.4. Health Insurance System

Since the launch of CEBHI, there has been a phenomenal increase in health insurance coverage; the enrolment of workers in the program has increased from about 4.8 million in 2008 to 12.01 million in 2017 [28]. The prompt execution of the ‘Saudization policy’ in private establishments has resulted in a massive increase in the percentage of Saudi nationals in the private sector workforce. The number of insured Saudis in the program increased from 0.45 million in 2008 to 3.44 million in 2020 [72]. Currently, private health insurance covers about 30% of the working population across the country through 28 licensed insurers [28]. The voluntary health insurance offered by these insurers contributes about 2.8% of total healthcare expenditure in KSA [70].

The insurance market in the KSA is the second largest in the GCC countries after the UAE, with a Gross Written Premium (GWP) of SR 42.03 billion (US$ 11.20) in 2021, and this market has grown in recent periods due to economic development and compulsory coverage [83]. However, due to adverse effects on employment during the COVID-19 pandemic, insurance penetration declined from 1.5% in 2020 to 1.3% in 2022, which is comparatively lower than the global average decline of 7.3% [84]. The low insurance penetration may be partly due to low awareness and demand for insurance, even though measures were introduced to improve community awareness while improving the efficiency of the insurance system [84].

### 3.4. Flow of Healthcare Funds to Providers of Care

The flow of healthcare funds to various healthcare providers (Table 2) shows that the government, health insurance, and households together contributed 68% of funds for hospital services, while 18% was spent in clinics delivering ambulatory healthcare. Unlike healthcare systems in other high-income countries, there is a disproportionate concentration of services in hospitals, resulting in competition for available funds between hospitals and primary healthcare centers.

The analysis further showed that 79% of public healthcare funding is being utilized by government hospitals [70]. This contrasts with the OECD benchmark average of 39%, and countries like Germany and Mexico, where less than 30% of health spending is directed towards hospital care [76]. This finding should be viewed in the current perspective of primary healthcare utilization in the KSA, as it is crucial to ensure the effective utilization of primary and preventive services by the population in the context of the increasing burden of lifestyle diseases.

### 3.5. Flow of Healthcare Funds to Healthcare Activities

The distribution of healthcare funds to various health activities shows that curative care, which includes outpatient and daycare services, accounted for 73.9% of healthcare expenditure in 2018 (Table 3). However, preventive care received only 3.8% of the total healthcare funds.

Prevention of diseases is one of the strategic goals under Vision 2030 [31]. However, only 5% of government healthcare expenditure is devoted to preventive care, which is significantly below the average of 8% for Asia-Pacific countries [70]. In contrast, developed countries such as the United Kingdom (12%) and Denmark (9%) allocate a larger portion of their health budgets to preventive care [12]. Evidence suggests that every dollar invested in effective public health programs at the community level can yield future savings of about US$ 5.6 in healthcare costs [85]. In the KSA, preventive care is offered in hospitals as well as primary healthcare facilities, with both establishments vying for financial resources to support primary care services. At present, there is a lack of adequate funding dedicated to preventing NCDs. With minimal funds allocated for preventing lifestyle diseases, it is expected that there will be a notable rise in costs to treat chronic diseases in the future.

### 3.6. Equity Analysis

While the KSA has made significant strides in reforming its healthcare financing system, certain equity concerns persist. Recent research that reviewed strategies and policy documents addressing equity perspectives suggested that none of the measures communicated an explicit focus on promoting health equity [86]. The healthcare system in the KSA offers free services to its citizens through government healthcare facilities. However, households often utilize private healthcare facilities due to quality and access issues. Studies have shown that people in low-income groups often face financial challenges due to OOPE when accessing private healthcare services. A recent survey showed that 50% of households used their current income to pay for healthcare services, with only 9% relying on health insurance [87].

While government employees and high-income workers in the private sector have access to health insurance, low-income groups in the private sector face limited coverage options [88,89]. Further, expatriates, who constitute a significant share of private sector workers, often have insurance plans that vary in quality, and many enrolled in basic plans may not receive comprehensive healthcare services [88]. The access to healthcare services for expatriate workers depends on their status of employment and the insurance plans offered by their employers.

Health insurance plays a significant role in reducing households’ OOPE on healthcare. For instance, one study found that health insurance reduces OOPE for healthcare services by 2% and for medicines by 2.4% [45]. However, its effectiveness in reducing OOPE varies across income groups. Another study showed that households in lower-income groups tend to spend a relatively higher proportion of their income compared to high-income groups [44]. Households with members suffering from chronic illnesses incur higher OOPEs compared to those without chronic conditions [46]. A recent study showed a 2.6% gap in relative OOPE among chronically ill and non-chronically ill households [43].

There are geographical disparities in health insurance coverage in urban and rural areas of the country. Households’ OOPE on healthcare services may vary across regions due to differences in the availability of healthcare services, the economic status of the region, and health insurance coverage rates. Health regions such as Jeddah and Riyadh have more access to healthcare infrastructure and better health insurance coverage compared to other regions [90]. Similarly, people in less populated regions may face challenges in obtaining healthcare services compared to highly populated regions [88]. These issues need to be addressed to enhance the financial protection ensured by health insurance. Studies have also shown significant differences in access and utilization of primary healthcare services among health regions.

## 4. Discussion

Health expenditure in the KSA has increased both in absolute terms and as a share of the GDP. In terms of GDP, the KSA’s health expenditure increased from 4.2% in 2000 to 5.97% in 2021 [20]. Even though the overall expenditure on healthcare has risen, the increase is significantly lower when compared to other high-income nations. The lower per capita healthcare expenditure is also reflected in health indicators such as the density of the health workforce, hospital beds, and other health resources [78]. For instance, the number of physicians in the KSA, 26.1 per 10,000 of the population, is lower compared to the United Kingdom (58.2), Germany (43), and Australia (37.6); and the number of nursing staff including midwives, at 58.2 per 10,000 of the population, is much lower compared to countries like the United States (156.9), Germany (134.9), and Australia (132.4) [5]. The number of hospital beds per 10,000 of the population in the KSA was 22.4 in 2021, which is low compared to countries such as the Russian Federation (71.2), China (43.1), and Australia (38.4) [1,5]. This implies the need for massive investment to increase healthcare resource availability and bring the Kingdom up to par with other high-income countries.

Evidence indicates that a higher density of physicians has a positive correlation with health outcomes [91,92], healthcare accessibility [93,94,95], and healthcare utilization [96]. Research has shown that a 10% increase in physician density is associated with a 2.3% improvement in healthcare accessibility and quality as measured by the Healthcare Access and Quality Index [96]. A significant association between physician density and healthcare utilization and health outcomes was consistently observed in other studies [97]. Studies have demonstrated that hospital bed capacity plays a significant role in improving health outcomes [98], quality of care [99], healthcare utilization [99,100,101], and access to healthcare [102]. The limited availability of hospital beds can lead to delayed treatments, overcrowding, high mortality rates, and limited access to inpatient care and preventive and acute healthcare services. This underlines the significance of policies aimed at increasing the supply of physicians in the workforce to improve health outcomes, healthcare utilization, and healthcare equity.

Due to overburdened public healthcare services, many people seek health services from the private sector, leading to OOPE. Many studies have shown that households with low income and chronic illnesses are spending a higher proportion of their income on OOPE for healthcare [103,104,105,106,107,108]. While the KSA provides universal access to healthcare for its citizens, there are still significant financial burdens, particularly for low-income households and those with chronic illnesses. The present healthcare financing system is insufficient in cushioning the chronically ill and the poor. A recent survey shows that households with the lowest income are the most likely to have to sell their belongings (3%) or ask for financial support from friends and family (7%) to cover healthcare expenses [84]. The expatriate workers employed in the private sector still incur significant OOPE on healthcare due to co-payments, limited coverage, and gaps in health insurance knowledge [23,24,25,26,27]. The extent of these expenditures can vary based on individual circumstances and the specific insurance plan provided.

Experiences in countries such as Japan and Korea show that employer-sponsored health insurance could mitigate OOPE on healthcare and reduce the financial burden of households [109,110]. In Switzerland, employer-based health insurance and mandatory individual insurance has helped to reduce inequities [111]. The introduction of multiple health insurance in Thailand has led to a significant decrease in health impoverishment, as well as a marked improvement in healthcare quality and equity [112]. Even the GCC countries such as Oman and Qatar spend a lower share on OOPE for healthcare. The Oman healthcare system is largely financed by the government (87.19% of CHE), with OOPE accounting for 6.13% of the CHE [113]. In Qatar, the government covers 90% of the total cost of healthcare, and OOPE constitutes 5.7%, one of the lowest in the region [19]. The variations in OOPE on healthcare demonstrated the need for diverse approaches to healthcare financing required to reduce household burden on healthcare expenditure.

The current pattern of healthcare expenditure in the KSA reveals a historical emphasis on curative care. While the exact share of the MOH budget allocated to primary healthcare in the KSA is unknown, it is believed that primary healthcare centers have been insufficiently funded [114]. OECD countries typically allocate about 13% of their health budgets to primary healthcare, with countries like Australia and Poland dedicating a larger share, approximately 17%, to primary healthcare [76]. Due to inadequate budget allocation, the primary healthcare system in the KSA is facing many challenges, including a limited local workforce, funding issues, and ineffective information system implementation [108]. Furthermore, the primary healthcare system in the KSA still faces a 40% deficiency in the number of physicians compared to its requirements [108]. It is suggested that allocating funds evenly between hospitals and primary care institutions can lead to increased value in healthcare investments [12]. Evidence suggests that nations that allocate significant resources to primary healthcare often produce better health outcomes, such as reduced mortality rates and increased life expectancy rates [109,110,115,116]. According to the WHO, scaling up various primary healthcare interventions in low- and middle-income economies could save 60 million lives and increase the average life expectancy by 3.7 years by 2030 [12]. Recognizing these benefits, Saudi Vision 2030 includes reforms aimed at improving resource allocation to primary healthcare and preventive services. These reforms are intended to reduce the dependency on curative care and improve health outcomes. In addition to the need for fair distribution across various levels of care, it is also imperative to enhance hospital efficiency by reducing waiting times, delivering effective treatment, and enhancing patient outcomes, which can ultimately result in cost savings and more efficient resource management [117,118,119].

Despite the inclusion of preventive care in the benefit packages of CEBHI, it is unlikely that insurance organizations will take an interest in investing in preventive programs intended to reduce the future costs of care. In countries such as South Korea, employer-based health insurance through NHI increased the utilization of preventive care [120]. NHI in Taiwan emphasizes preventive programs, including annual health check-ups, which has lead to higher satisfaction and improved health outcomes among the insured [121]. These experiences underscore the importance of developing more inclusive health insurance policies that provide preventive care to all citizens and expatriates, regardless of their employment status. It is high time that the government develops and implements sustainable preventive care strategies involving all stakeholders such as MOH, other ministries, the private health sector, and civil society for mandatory implementation. Moreover, it is important to orient both insurance companies and employers on the significance of prevention strategy and provide them with the necessary guidelines for its implementation.

Determining the appropriate portion of a country’s healthcare budget to allocate for personnel compensation is essential for the efficient provision of healthcare services. While there are no widely agreed-upon criteria, it is imperative to ensure that there is sufficient funding for staff to maintain the quality of services. The MOH budget allocation focuses heavily on personnel compensation, potentially reducing the available budget for purchasing medications and other essential healthcare supplies. The growth in personnel compensation is likely due to the increasing proportion of Saudi nationals in the national healthcare workforce. The IMF (2020) data reveal that the KSA dedicates a substantial proportion of its general government expenditure (53.73%) towards personnel compensation, outpacing other countries globally [122]. Substantial personnel costs significantly affect the government’s budget, impacting spending efficiency and value for money. Therefore, the government must introduce strategies and mechanisms to efficiently control personnel costs, guaranteeing fiscal stability and optimal resource allocation. This could involve overseeing salary budgets, managing headcount levels, closely tracking budgeted positions, preparing for anticipated vacancies in the salary budget, determining optimal staffing levels based on need, and benchmarking with other countries of similar development.

There has been concern about how to sustain the current free healthcare services and the government’s ability to meet the growing demand for healthcare, especially when revenues from oil resources are uncertain. As part of NTP, the country has introduced various taxes to reduce its oil dependency and increase non-oil revenues [123]. The KSA has levied taxes on goods and services, corporate income, withholding, capital gains, and customs duties, including ‘Zakat’, a compulsory tax levied on individual assets. The revenues from these taxes were projected to reach SR 315 billion (US$ 84 billion) by February 2022 [123]. Although the Kingdom imposed high taxes on tobacco products (between 2013 and 2019, the tax on cigarettes increased by 175.21%), the revenue mobilized from these taxes is not allocated for any specific health-related activities [124]. Key objectives of Vision 2030 are to reduce the reliance on oil as a revenue source for all economic sectors, including health, and to reduce public spending with a greater focus on private sector involvement. Recent years witnessed a surge in private investment in non-oil sectors and, as a result, the contribution of the non-oil economy to GDP has increased significantly. According to a recent estimate, non-oil activities constituted about 50% of Saudi Arabia’s real GDP in 2023 [125].

Administration expenses related to health insurance schemes can considerably influence the premiums. Most of the OECD countries regulate the administration of health insurance companies and impose a cap on their profits [76]. The expenditure on administration of CEBHI accounts for 18.7% of total expenditure, which is higher than many other countries [53]. Countries with relatively high coverage of mandatory health insurance, such as the USA (12%), Switzerland (5%), and the Netherlands (3%), have lower administration costs, effectively using their premiums to provide healthcare services to the target population [70]. The Council for Health Insurance (CHI) should ensure the efficient operation of CEBHI, achieving the goal of cost-shifting and enhancing insured individuals’ access to high-quality healthcare services. By implementing strategies such as predictive analytics, AI technologies for claims processing, and promoting telemedicine and virtual care options, insurers can significantly reduce administrative costs.

There has been a consistent increase in the premiums charged by insurers. Health insurance premiums are driven by factors such as policy features, entitlements, insurers’ strategies, and cost structures. A rise in healthcare costs in the private sector has also been attributed to the implementation of a 5% VAT in 2018, which was increase to 15% in May 2020 [41]. The cost of private sector care is projected to increase further as a result of the VAT, which will have a considerable impact on high-risk expatriate workers. Currently, there is no reliable system in place to ensure that insurance companies provide coverage to high-risk workers in the private sector. The risk-rated premiums that health insurance companies currently adopt are comparable to voluntary or private health insurance premiums. Moreover, premiums on health insurance are likely to go up due to the inclusion of VAT in most medical goods and services.

Evidence suggests that efficiency in healthcare system financing can be improved by increasing the share of public healthcare funding and avoiding the fragmentation of financing through implementing a universal health insurance (UHI) system. This approach has proven effective in countries like Germany, Canada, and France, which have efficient healthcare systems [126]. Saudi Vision 2030 aims to implement a national health insurance (NHI) system involving the private sector to reduce dependence on government budgets and improve the quality and accessibility of healthcare services. Health insurance programs in low- and middle-income countries have been found to improve access to healthcare, as measured by increased utilization of healthcare facilities and improved financial protection [127,128]. NHI experiences in Ghana [129], Taiwan [130], and Vietnam [131] suggest that NHI can decrease OOPE on healthcare and improve access to services. In Zambia, NHI has the potential to improve financial resources for healthcare, access to high-cost intervention, and patient satisfaction [132].

Health policymakers are seeking ways to introduce a sustainable health services delivery system by implementing a contributory NHI that is based on regular contributions from the people [111]. Several studies have explored the viability and acceptability of health insurance programs by assessing the willingness to pay (WTP) for an NHI by various segments of the population [56,57,58,59,60,61]. Studies have elicited the WTP for improved access to health services that are presently delivered free of cost by the MOH [56,59]. These studies have shown that nearly two-thirds of the respondents were willing to contribute towards quality improvements in services delivered by government healthcare facilities. Further, few studies based on national survey data reveal that people who own health insurance policies are more likely to demand preventive check-ups, which helps them to lower future costs of medical treatment [52]. Studies also suggest that health insurance also helped people to lower OOPE and medicine expenditure [45]. These findings suggest that expanding access to health insurance throughout the Kingdom is likely to facilitate improvement in health outcomes and lessen households’ OOPE on healthcare.

The healthcare landscape in the KSA will continue to evolve as the government pursues reforms in healthcare financing and delivery. Enhancing privatization initiatives in healthcare remains an essential part of the KSA’s economic diversification efforts, intended to relieve government of the increasing financial burden of delivering free healthcare to its people [30,133]. The focus of Saudi Vision 2030 is on the privatization of healthcare, direct foreign investment in the healthcare industry, and public–private partnerships which are anticipated to have a large impact on how healthcare is funded and delivered. Under Vision 2030, the KSA aims to allocate more than $65 billion towards enhancing the healthcare infrastructure, restructuring and privatizing healthcare services and insurance, establishing 21 “health clusters” across the country, and increasing access to e-health services. Moreover, it seeks to raise private sector involvement from 40 to 65% by 2030, with plans to privatize 290 hospitals and 2300 primary health centers, presenting substantial business opportunities for foreign companies in the development of the KSA’s healthcare sector [65]. The goal is to improve healthcare access, equity, efficiency, and quality of care while reducing the financial burden of the government. To facilitate the privatization process, the Kingdom enacted the private sector participation law in March 2021. This law regulates the environment in which privatization projects and public–private partnerships are carried out in the KSA [133]. Given the increased private sector contribution, growing health insurance market, and rising public spending, the KSA’s healthcare expenditure is expected to increase significantly.

Saudi Vision 2030 also emphasizes tackling the growing burden of NCDs by strengthening the primary healthcare system, which is the core of the new healthcare delivery model. The patient-centered healthcare delivery model, organized into clusters, aims to prioritize the provision of preventive services and is likely to reduce public healthcare expenditure, particularly on hospital care. Finally, the gradual advance in health sector reforms and the government’s policy commitment to develop a robust primary healthcare network is expected to play a significant role in controlling the rapid growth of public healthcare expenditure in Saudi Arabia.

## 5. Policy Recommendations

The present review suggests that the expansion of health insurance coverage and enhancing resource allocation to primary care is critical for the sustainability of the healthcare financing system in the KSA. Moreover, strategies and policies that integrate public and private funding to ensure equitable health insurance coverage according to the health needs of the population are necessary. The healthcare financing system in the KSA is inadequate in cushioning the chronically ill and the poor. This calls for integrating a social safety net into the existing healthcare financing mechanisms, including CEBHI, to prioritize the poor and chronically ill. Furthermore, the CEBHI program should expand its coverage to include small businesses, low-income individuals, and high-risk workers, thereby ensuring better access to healthcare services and financial protection. A higher allocation to primary care services is crucial in tackling existing healthcare problems, including control of NCDs, and achieving the nation’s healthcare goals. This shift in focus can lead to a more sustainable, efficient, and equitable healthcare system that is better equipped to meet the evolving healthcare needs of the population.

Additionally, policymakers need to prioritize implementing new regulations to promote preventive healthcare strategies in all areas of the healthcare system. The Kingdom should introduce appropriate strategies and mechanisms to efficiently control personnel costs and optimize resource allocation in healthcare. This may include determining optimal staffing levels based on needs and benchmarking with other countries of similar development. Finally, massive investment and reforms will be necessary to bring the Saudi healthcare system fully on par with other high-income nations. The success of these initiatives will depend on the effective implementation of privatization, public–private partnership arrangements, and the ability to adapt to the changing healthcare needs of the population.

## 6. Future Research

There is limited information on the allocation and expenditures of various healthcare programs and activities, as well as the equity implications of different healthcare financing mechanisms. This gap hinders effective policy analysis and discussions on healthcare financing. Future research should focus on the allocation and expenditure of health programs, equity in financing, and the delivery of healthcare services. Studies should also explore the costs of public and private healthcare services, and examine the impact of OOPE on healthcare among different income groups, including the poor, marginalized, older age groups, and expatriates. It is also necessary to study the cost-effectiveness of various preventative interventions and the impact of various funding models on service delivery and provider behavior. Further, studies are required on the effectiveness of the CEBHI program in expanding coverage and reducing household OOPE on healthcare. Furthermore, future research should focus on the impact of privatization and public–private partnership initiatives on improving healthcare access, quality of care, and equity. These studies can provide valuable insights to guide healthcare financing policies and reforms in the KSA as it works towards achieving its Vision 2030 goals.

## 7. Conclusions

This comprehensive review shows that there has been a significant increase in health expenditure in the KSA over the past decade; however, the increase remains modest compared to other high-income nations. Despite implementing significant reforms in its healthcare system, the share of private healthcare expenditure in the Kingdom’s current healthcare expenditure has seen only marginal growth. Nevertheless, the private health sector is likely to play a significant role in healthcare financing and service provision, with widespread coverage of the population under health insurance. The healthcare financing system faces several challenges, including demographic transitions, an aging population, increasing NCDs, and the increasing demand for quality healthcare. Moreover, inefficiencies in resource allocation persist due to the disproportionate focus on hospital services over primary care in the government health budget. The current healthcare financing system appears insufficient to provide adequate support for the chronically ill and the poor. Finally, government healthcare expenditure devoted to primary and preventive care is significantly below the average for Asia-Pacific countries. Therefore, it is high time that the government allocates more resources for primary care and implements sustainable preventive care strategies involving all stakeholders, including the MOH, other government ministries, the private health sector, insurance companies, and civil society, to ensure mandatory implementation.

## Figures and Tables

**Table 1:** Budget allocated to MOH and expenditure in percentage—2010–2023 (in Million Riyal).

Year	Total State Budget	MOH Budget	MOH Expenditure as a Share of State Budget
2010	540,000	35,063	6.50%
2011	580,000	35,063	6.90%
2012	690,000	47,076	6.80%
2013	820,000	54,350	7.00%
2014	855,000	59,985	7.02%
2015	860,000	62,342	7.20%
2016	840,000	58,899	7.01%
2017	890,000	67,758	7.61%
2018	978,000	64,297	6.60%
2019	1,106,000	74,403	6.82%
2020	1,020,000	75,413	7.40%
2021	990,000	79,849	7.82%
2022	955,000	77,696	8.00%
2023	1,114,000	80,752	7.00%

Source: Ministry of Health, KSA. Statistical Yearbook, Various Years.

**Table 2:** Flow of healthcare funds to healthcare providers in the KSA (2018) (in billion Saudi Riyal).

Healthcare Financing Schemes	Government Schemes	Compulsory Contributory Health Insurance	Voluntary Healthcare Payment	Household OOPE	All Health Financing Schemes
Hospitals	93.75 (78.65%)	12.16 (59.26%)	0.41 (14.19%)	8.89 (32.96%)	115.30 (68.0%)
Ambulatory healthcare providers	15.62 (13.10%)	2.31 (11.25%)	2.23 (77.16%)	10.65 (39.5%)	30.82 (18.18%)
Ancillary healthcare providers	1.81 (1.52%)	0.00	0.00	0.02 (0.07%)	1.83 (1.08%)
Retailers and other suppliers of medical goods	0.00	2.21 (10.77%)	0.24 (8.30%)	7.24 (26.85%)	9.69 (5.72%)
Preventive care providers	2.04 (1.71%)	0.00	0.00	0.00	2.04 (1.20%)
Health system administration	3.85 (3.23%)	3.83 (18.66%)	0.00	0.00	7.68 (4.53%)
Rest of the world	2.13 (1.78%)	0.00	0.00	0.07 (0.26%)	2.20 (1.23%)
All healthcare providers (in billion Saudi Riyal)	119.19 (100%)	20.52 (100%)	2.89 (100%)	26.96 (100%)	169.55 (100%)

Source: compiled from National Health Accounts Estimates 2020, Saudi Health Council. Note: figures in parentheses show the percentage of funds distributed by each financing scheme to different healthcare providers, including administration.

**Table 3:** Flow of healthcare funds to healthcare activities in the KSA (in billion Saudi Riyal).

Financing Schemes	Government	Compulsory Health Insurance	Voluntary Health Insurance	Household OOPE	All Health Financing Schemes
Curative care (inpatient, outpatient, and daycare)	92.22 (77.37%)	14.21 (69.25%)	1.05 (36.33%)	17.80 (66.02%)	125.28 (73.89)
Rehabilitative care	0.07 (0.05%)	0.17 (0.83%)	0.71 (24.56%)	0.71 (26.33%)	1.25 (0.74)
Ancillary services (labs, imaging, and patient transport)	1.81 (1.52%)	0.00	0.36 (12.46%)	0.02 (0.07%)	2.18 (1.29)
Medical goods	15.25 (12.80%)	2.31 (11.26%)	0.41 (14.18%)	8.12 (30.12%)	26.09 (15.39)
Preventive care	5.99 (5.02%)	0.00 00	0.35 (12.11%)	0.11 (0.41%)	6.45 (3.80)
Health system governance	3.85 (3.23%)	3.83 (18.66%)	0.00	0.00	7.68 (4.53)
Other healthcare services	0.00	0.00	0.00	0.62 (2.30%)	0.62 (0.37)
All healthcare functions (in billions)	119.19 SAR (100%)	20.52 (100%)	2.89 (100%)	26.96 (100%)	169.55 (100%)

Source: compiled from National Health Accounts estimates 2020, Saudi Health Council. Note: figures in parentheses show the percentage of expenditures incurred on different healthcare activities by each financing scheme.

## Data Availability

Available on request from the authors.
